# Imatinib dose escalation versus sunitinib as a second-line treatment against advanced gastrointestinal stromal tumors: A nationwide population-based cohort study

**DOI:** 10.18632/oncotarget.16795

**Published:** 2017-04-03

**Authors:** Jun-Te Hsu, Puo-Hsien Le, Chang-Fu Kuo, Meng-Jiun Chiou, Chia-Jung Kuo, Tsung-Hsing Chen, Chun-Jung Lin, Jen-Shi Chen, Huang-Pin Yu, Chun-Nan Yeh, Yi-Yin Jan, Ta-Sen Yeh

**Affiliations:** ^1^ Department of Surgery, Chang Gung Memorial Hospital at Linkou, Chang Gung University College of Medicine, Taoyuan, Taiwan; ^2^ Department of Gastroenterology, Chang Gung Memorial Hospital at Linkou, Chang Gung University College of Medicine, Taoyuan, Taiwan; ^3^ Division of Rheumatology, Allergy, and Immunology, Chang Gung Memorial Hospital at Linkou, Chang Gung University College of Medicine, Taoyuan, Taiwan; ^4^ Office for Big Data Research, Chang Gung Memorial Hospital at Linkou, Taoyuan, Taiwan; ^5^ Department of Hemato-Oncology, Chang Gung Memorial Hospital at Linkou, Chang Gung University College of Medicine, Taoyuan, Taiwan; ^6^ Department of Anesthesiology, Chang Gung Memorial Hospital at Linkou, Chang Gung University College of Medicine, Taoyuan, Taiwan

**Keywords:** gastrointestinal stromal tumor, imatinib, sunitinib, dose escalation, survival

## Abstract

**Background:**

Although treatment with imatinib in advanced gastrointestinal stromal tumor (GIST) patients has led to significant clinical benefits, the disease will eventually progress due to imatinib resistance. Treatment options after failure of first-line imatinib include imatinib dose escalation or shifting to sunitinib. However, there is no large-scale study to compare the efficacy difference between these two treatment strategies or the role of surgery.

**Results:**

This study recruited 521 advanced GIST patients including 246, 125, and 150 placed in groups 1, 2, and 3, respectively. Groups 1 and 2 had significantly longer overall survival (OS) as compared with the group 3 (median 37.5 months versus 16.0 months; *p* < 0.0001). After adjusting for confounding variables, groups 1 and 2 had longer OS than group 3. A favorable survival trend was seen with surgery, although this benefit disappeared after adjusting for confounding factors.

**Materials and Methods:**

We conducted a nationwide population-based cohort study using data from the Taiwan National Health Insurance Research Database from July 2004 to December 2010. Advanced GIST patients who no longer responded to first-line imatinib were stratified into three groups: imatinib dose escalation (group 1); imatinib dose escalation and a shift to sunitinib (group 2); a direct shift to sunitinib (group 3). The therapeutic success of the three treatment regimens and the effect of surgery were evaluated by overall survival.

**Conclusions:**

For advanced GIST patients who failed first-line imatinib treatment, imatinib dose escalation confers significantly longer OS compared to a direct switch to sunitinib. Surgery does not provide survival benefits.

## INTRODUCTION

Gastrointestinal stromal tumors (GISTs) account for 0.1–0.3% of gastrointestinal malignant neoplasms and are found mainly in the mesenchyme of the gastrointestinal tract [1]. They can occur at any age and have a patient median age of 60-65 years, typically causing bleeding, anemia, and abdominal pain. Approximately 50-60% of GISTs occur in the stomach, followed by the small bowel (20–30%), and less frequently the colon and rectum (5%) and esophagus (< 1%) [2]. About 15% of GIST patients are diagnosed with metastatic disease [3]. Treatment of advanced GIST patients with imatinib has led to significant clinical benefits not only in prolonging the median progression-free survival (PFS) but also in extending overall survival (OS) [4, 5, 6]. Nonetheless, the disease eventually progresses due to imatinib resistance. Major mechanisms proposed to cause acquired imatinib resistance include mutations in the mechanisms proposed to cause acquired imatinib resistance include mutations in the KIT or platelet-derived growth factor receptor-alpha (PDGFRA) genes, KIT or PDGFRA gene amplification, and activation of alternative pathways [7].

About 85–95% of GISTs have been identified to harbor gain-of-function mutations in the KIT gene, with 85% of these mutations occurring in exon 11 and 15% in exon 9. Another activating mutation occurs in the PDGFRA gene, mainly affecting exon 18 [8, 9]. The severity of GISTs is determined by their primary location, tumor size, and mitosis count from histological examinations [10]. There was no effective medicine treatment for GISTs before 2001 [4].

Treatment options for advanced GIST after failure of first-line imatinib treatment include imatinib dose escalation or shifting to sunitinib. Other local ablative therapies involve surgical resection, radiofrequency ablation, trans-arterial chemoembolization, and radiotherapy. Demetri et al. suggested in a randomized controlled trial that advanced GIST patients with failure of imatinib or intolerance to imatinib treatment should switch to sunitinib [11]. Chen et al. also supported the same treatment strategies in those situations [12]. Recently, Vincenzi et al. indicated that a second-line treatment with sunitinib was associated with an improvement median time to progression in KIT exon 11 mutated patients progressing from imatinib 400 mg/day [13]. In contrast, two phase III studies have shown that imatinib dose escalation is an alternative in treating advanced GIST patients [14, 15]. A European Organization for Research and Treatment of Cancer study demonstrated that the partial response and stable disease rates were 2% and 27%, respectively, after imatinib dose escalation to 800 mg/day [14]. Another trial also reported similar results with a partial response rate of 7% and a stable disease rate of 32% following imatinib dose escalation to 800 mg/day [15]. Furthermore, Hsu et al. suggested that comparable results were achieved with imatinib dose escalation or sunitinib treatment after failure of first-line imatinib in advanced GIST patients [16]. In addition, a meta-analysis of 1,640 patients with advanced GIST demonstrated that patients with KIT exon 9 mutations who were treated with high-dose imatinib exhibited significantly longer PFS and higher objective response rates than treated with standard dose [17]. Since previous studies recruited very few advanced GIST patients treated with imatinib dose escalation or sunitinib and there are no randomized controlled trials testing the effectiveness of the two different treatment strategies, we conducted a nationwide population-based study in Taiwan to compare the therapeutic effects on advanced GIST patients treated with imatinib dose escalation or a direct shift to sunitinib. We also explored the impact of surgery on survival in advanced GIST patients with failure of first-line imatinib treatment.

## RESULTS

**Figure 1:**
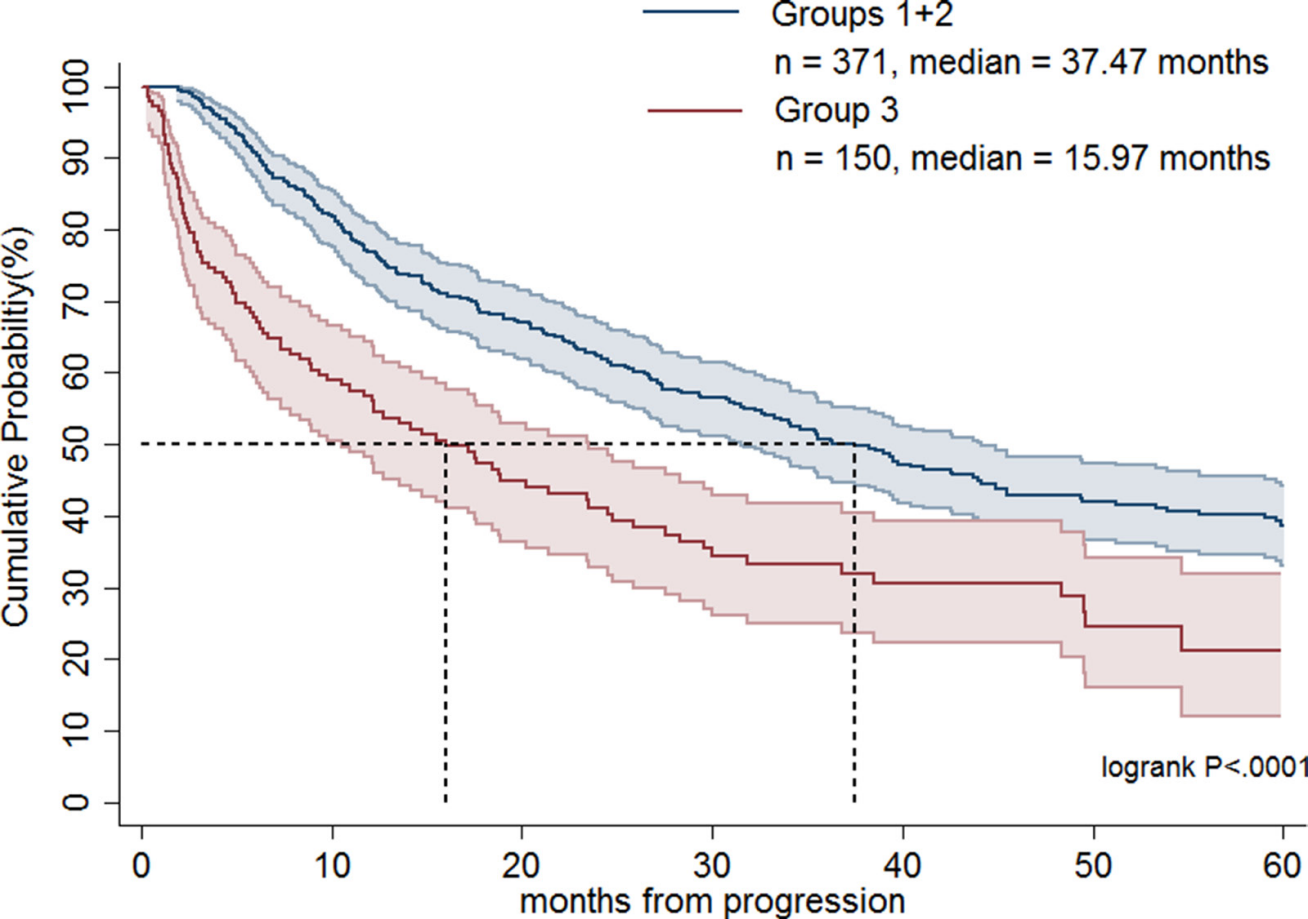
Overall survival rates of advanced gastrointestinal stromal tumor patients treated with imatinib dose escalation (group 1) plus imatinib dose escalation followed by sunitinib (group 2) and a direct switch to sunitinib (group 3)

**Figure 2:**
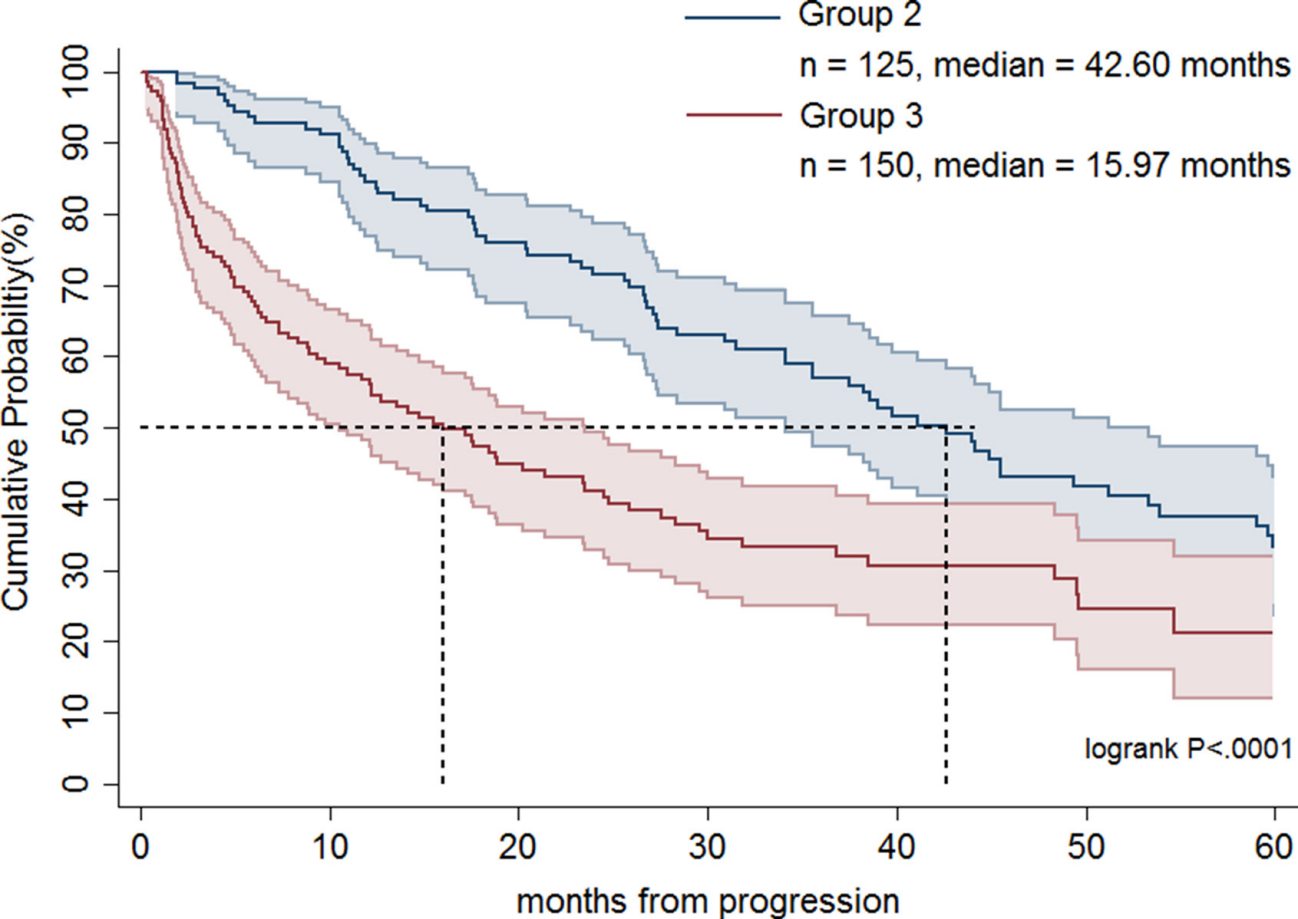
Overall survival rates of advanced gastrointestinal stromal tumor patients treated with imatinib dose escalation followed by sunitinib (group 2) and a direct switch to sunitinib (group 3)

**Figure 3:**
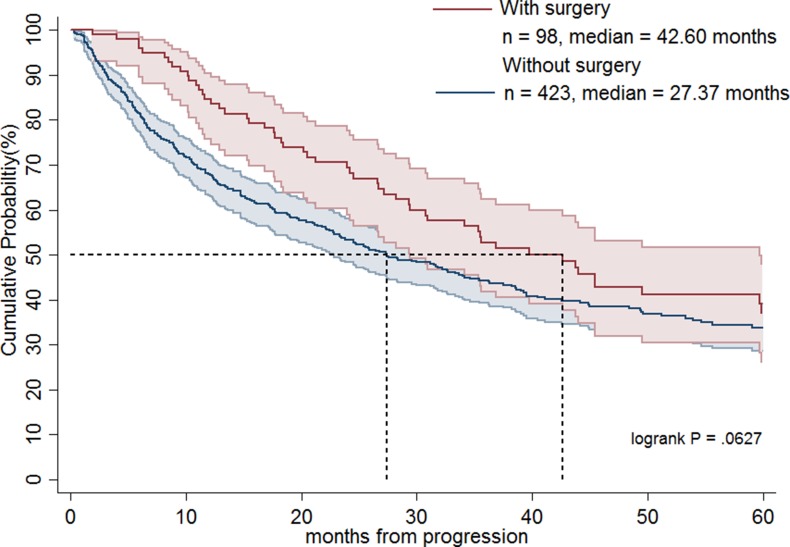
Overall survival rates of advanced gastrointestinal stromal tumor patients treated with imatinib dose escalation (group 1), imatinib dose escalation followed by sunitinib (group 2) and a direct switch to sunitinib (group 3) in terms of surgery

**Figure 4:**
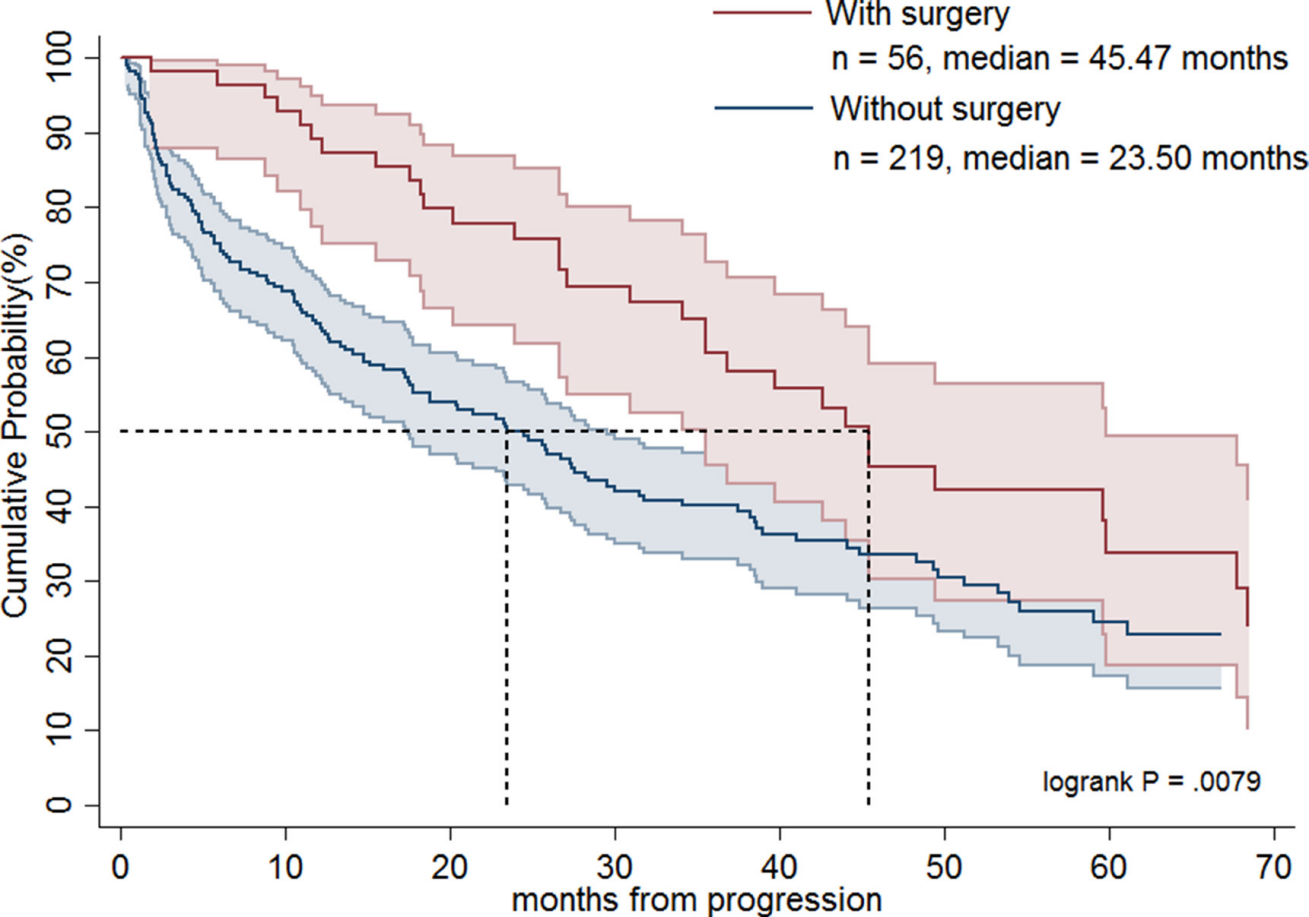
Overall survival rates of advanced gastrointestinal stromal tumor patients treated with imatinib dose escalation followed by sunitinib (group 2) and a direct switch to sunitinib (group 3) in terms of surgery

Table 1 shows the demographics of the three treatment groups. Patient age was significantly higher in group 3 compared with groups 1 and 2 (*p* < 0.0001). The largest percentages of Charlson comorbidity index (> 3) were noted in group 3 (*p* = 0.0297). Group 2 had a greater proportion of patients undergoing surgery than the other two groups (*p* < 0.0001). There was no significant difference in sex or place of residence among groups. As shown in Figure 1, groups 1 and 2 had significantly longer OS than group 3 (median, 37.5 months versus 16.0 months; *p* < 0.0001). Table 2 demonstrates that patients in groups 1 and 2 had a statistically significant better prognosis than those in group 3 as calculated by models adjusting for confounding factors including age, sex, and operation or age, sex, Charlson comorbidity index score, and operation. Favorable OS was noted for group 2 patients compared to group 3 patients (Figure 2; median = 42.6 months versus 16.0 months, respectively; *p* < 0.0001). However, after adjusting for confounding factors in different models, groups 2 and 3 patients had comparable outcomes (Table 2). Figure 3 shows that patients in groups 1, 2, and 3 treated with surgery had a trend towards longer OS as compared with those without (median, 42.6 months versus 27.4 months; *p =* 0.0627). Group 2 and 3 patients undergoing surgical treatment had a significantly more favorable prognosis than those not treated with surgery (Figure 4; median = 45.5 months versus 23.5 months; *p =* 0.0079). The benefit of surgery on OS disappeared after adjusting for confounding factors (Table 3).

**Table 1:** Demographics of advanced gastrointestinal stromal tumor (GIST) patients with a failure of first-line imatinib treatment

	GIST patients (*n* = 521)			
	Group 1 (*n* = 246)	Group 2 (*n* = 125)	Group 3 (*n* = 150)	*P* value
Sex				0.8424
Male	150 (60.98)	80 (64.00)	94 (62.67)	
Female	96 (39.02)	45 (36.00)	56 (37.33)	
Age (years) at progression				< 0.0001
Mean ± standard deviation	56.63 ± 16.23	58.75 ± 11.76	66.21 ± 13.05	
Place of residence, No. (%)				0.2260
Urban	60 (24.39)	40 (32.00)	33 (22.00)	
Suburban	71 (28.86)	33 (26.40)	42 (28.00)	
Rural	110 (44.72)	49 (39.20)	75 (50.00)	
Unknown	5 ( 2.03)	3 ( 2.40)	0 (0.00)	
Charlson comorbidity index				
Mean ± standard deviation	3.21 ± 2.13	3.12 ± 2.05	3.75 ± 2.54	0.0297
≤ 3	187 (76.02)	99 (79.20)	99 (66.00)	0.0267
> 3	59 (23.98)	26 (20.80)	51 (34.00)	
Ulcer disease	41 (16.67)	26 (20.80)	29 (19.33)	0.5894
Diabetes	30 (12.20)	16 (12.80)	31 (20.67)	0.0544
Chronic pulmonary disease	27 (10.98)	13 (10.40)	16 (10.67)	0.9851
Metastatic solid tumor	17 (6.91)	8 (6.40)	16 (10.67)	0.3162
Diabetes with end organ damage	15 (6.10)	5 (4.00)	13 (8.67)	0.2799
Cerebrovascular disease	17 (6.91)	2 (1.60)	10 (6.67)	0.0849
Congestive heart failure	5 (2.03)	5 (4.00)	11 (7.33)	0.0339
Connective tissue disease	9 (3.66)	3 (2.40)	2 (1.33)	0.3719
Mild liver disease	9 (3.66)	3 (2.40)	2 (1.33)	0.3719
Moderate or severe renal disease	7 (2.85)	2 (1.60)	13 (8.67)	0.0050
Peripheral vascular disease	5 (2.03)	3 (2.40)	6 (4.00)	0.4889
Operation				< 0.0001
Yes	42 (17.07)	42 (33.60)	14 (9.33)	
No	204 (82.93)	83 (66.40)	136 (90.67)	
Sunitinib dose (mg; cDDD)				< 0.0001
< 25		9 (7.20)	36 (24.00)	
> 25; < 37.5		78 (62.40)	78 (52.00)	
> 37.5; < 50		38 (30.40)	36 (24.00)	

Abbreviations: cDDD, cumulative defined daily dose.

**Table 2:** Overall survival rates of advanced gastrointestinal stromal tumor patients with a failure to first-line imatinib treatment in different adjusting models

Group	Total	Death (%)	Adjusted HR **(95% CI)**^a^	Adjusted HR **(95% CI)**^b^	Adjusted HR **(95% CI)**^c^
Groups 1+2	371	215 (57.95)	0.61 (0.47–0.78)*	0.61 (0.48–0.79)*	0.62 (0.48–0.79)*
Group 3	150	95 (63.33)	Reference	Reference	Reference
Group 2	125	75 (60.00)	1.31 (0.94–1.82)	1.33 (0.95–1.87)	1.33 (0.95–1.87)
Group 3	150	95 (55.88)	Reference	Reference	Reference

Abbreviations: HR, hazard ratio; CI, confidence interval.

^a^adjusted for age, sex.

^b^adjusted for age, sex, operation.

^c^adjusted for age, sex, Charlson comorbidity index, operation.

*p < 0.05.

**Table 3:** Overall survival rates of advanced gastrointestinal stromal tumor patients with a failure of first-line imatinib treatment in different adjusting models in terms of surgery

Group	Total	Death (%)	Adjusted HR (95% CI)^a^	Adjusted HR (95% CI)^b^	Adjusted HR (95% CI)^c^
Groups 1+2+3					
Yes	98	57 (58.16)	0.87 (0.65–1.17)	0.92 (0.68–1.24)	0.92 (0.69–1.24)
No	423	253 (59.81)	Reference	Reference	Reference
Groups 2+3					
Yes	56	32 (57.14)	0.98 (0.66–1.47)	0.90 (0.60–1.37)	0.90 (0.60–1.37)
No	219	138 (63.01)	Reference	Reference	Reference

Abbreviations: HR, hazard ratio; CI, confidence interval.

^a^adjusted for age, sex.

^b^adjusted for age, sex, drug use.

^c^adjusted for age, sex, Charlson comorbidity index, drug use.

## DISCUSSION

To the best of our knowledge, this large-scale nationwide population-based cohort study is the first to demonstrate that for advanced GIST patients who fail first-line imatinib treatment, imatinib dose escalation is associated with longer OS as compared with a direct shift to sunitinib, and that surgery does not provide survival benefits.

Currently, high-risk GIST patients or those patients faced with disease progression receive imatinib 400 mg/day as a standard adjuvant therapy or first-line therapy [6]. However, the second-line therapy in failure of imatinib treatment is still a matter of debate. There is little data and the studies that have been performed were not randomized and, therefore, are less trustworthy. Vincenzi et al. demonstrated that second-line therapy with sunitinib was associated with longer time to progression as compared with imatinib dose escalation in patients with exon 11 mutation (median = 10 months versus 5 months; *p* < 0.012). However, the OS did not differ between the two groups (median = 58 months versus 62 months; *p =* 0.883) [13]. Hsu et al. showed that imatinib dose escalation had similar PFS (median = 7.4 months versus 9.9 months; *p* < 0.316) and OS (median = 30.0 months versus 35.5 months; *p* < 0.599) as compared with a direct shift to sunitinib in treating advanced GIST patients who failed first-line imatinib [16]. In a subgroup analysis of patients without exon 9 mutation, sunitinib had significantly better PFS than imatinib dose escalation (median = 14.3 months versus 6.2 months; *p* < 0.037) but the OS was comparable between the two treatment groups [16]. Furthermore, Hislop et al. conducted a systemic review of evidence on the effectiveness of imatinib dose escalation or sunitinib for treatment of advanced GIST following progression with imatinib 400 mg/day [18]. They recommended imatinib dose escalation for advanced GIST after progression at standard 400 mg/day imatinib treatment, consistent with the treatment guidance proposed by others [19–21]. Our current study of 521 advanced GIST patients with failure of first-line imatinib treatment treated with imatinib dose escalation or a direct shift to sunitinib represents the largest published series worldwide, and indicates that imatinib dose escalation had significantly longer OS than a direct switch to sunitinib (median = 37.5 months versus 16.0 months; *p* < 0.0001). Nonetheless, a well-designed randomized study is required to help guide treatment strategies.

Recurrent, metastatic, and locally advanced GISTs can be treated successfully with imatinib [6, 17]. Although no randomized phase III trial has been performed to answer the question about its benefit, surgery for residual disease has been suggested in non-refractory metastatic GISTs to reduce the probability of resistant recurrent clones [22]. In this regard, Park et al. has shown that surgery prolongs PFS and OS as compared with non-surgery for recurrent or metastatic GIST patients who achieved a partial response or stable disease for more than 6 months with imatinib treatment [23]. Yeh et al. indicated that surgery may benefit select GIST patients with partial response, stable disease or local progression under imatinib treatment, especially for patients with local progression [24]. They also suggested that surgery may prevent the potential development of secondary mutations in select patients who responded to imatinib, as evidenced by the fact that secondary mutations were identified more frequently in GIST patients with local progression after surgery than those who responded [24]. Furthermore, Hsu et al. have shown that in comparison to non-surgery, surgery significantly prolonged PFS in patients with exon 9 mutation treated with imatinib dose escalation [16]. Interestingly, our present study indicated that surgery seems to prolong patient survival in groups 2 and 3. However, after adjusting for confounding variables, the beneficial effects of surgery were not evident. These findings suggest that surgery might benefit highly select patients who are good surgical candidates with favorable general performance status and less comorbidity. To answer whether surgical intervention may confer survival advantages in all advanced GIST patients who failed first-line imatinib treatment, a randomized controlled trial is needed.

Our previous study indicated that the most common adverse events were hematological in advanced GIST patients treated with imatinib dose escalation or sunitinib [16]. More than 60% of patients in both groups had anemia of any grade. Patients treated with sunitinib experienced higher percentages of leukopenia (60.7%), neutropenia (57.1%), and thrombocytopenia (57.1%) of any grade as compared to patients treated with imatinib dose escalation (27.0%, 27.0%, 17.5%, respectively). The most common non-hematological adverse events of any grade in patients treated with imatinib dose escalation were edema (23.8%) and bleeding (22.2%), while patients treated with sunitinib had diarrhea (50.0%), hand-foot syndrome (50.0%) and hypertension (50.0%). Nonetheless, most non-hematological adverse events were mild and manageable, with the most common grade 3 or 4 adverse event being hand-foot syndrome in 25% of patients treated with sunitinib. When determining treatment for GIST patients who no longer respond to first-line imatinib treatment, the side effects of second-line therapy including high-dose imatinib (> 400 mg/day) or sunitinib should be considered.

Although our present study suggests that patients treated with imatinib dose escalation had better OS compared with those switched directly to sunitinib, and that surgery did not provide survival advantages after failure of first-line imatinib treatment, there are several concerns and limitations inherent to this study. First, this is a retrospective study where patient selection could not be randomized. Second, there is lack of genetic information acquired in the nationwide database. Third, disease re-progression after second-line treatment could not be precisely evaluated based on the nationwide database without imaging data. Lastly, patients undergoing surgical interventions are affected by a selection bias including the physician’s judgment, patient’s general performance, concomitant diseases, and tumor-related complications. However, the strength of our current study is that its large-scale sample size covered a nationwide database whose statistical power is generally strong.

In conclusion, for advanced GIST patients who failed first-line imatinib treatment, imatinib dose escalation confers significantly longer OS as compared with a direct switch to sunitinib, and surgery does not provide survival benefits. A prospective randomized controlled study is recommended for validating the treatment strategy for advanced GIST patients with failure of first-line imatinib treatment.

## MATERIALS AND METHODS

**Figure 5:**
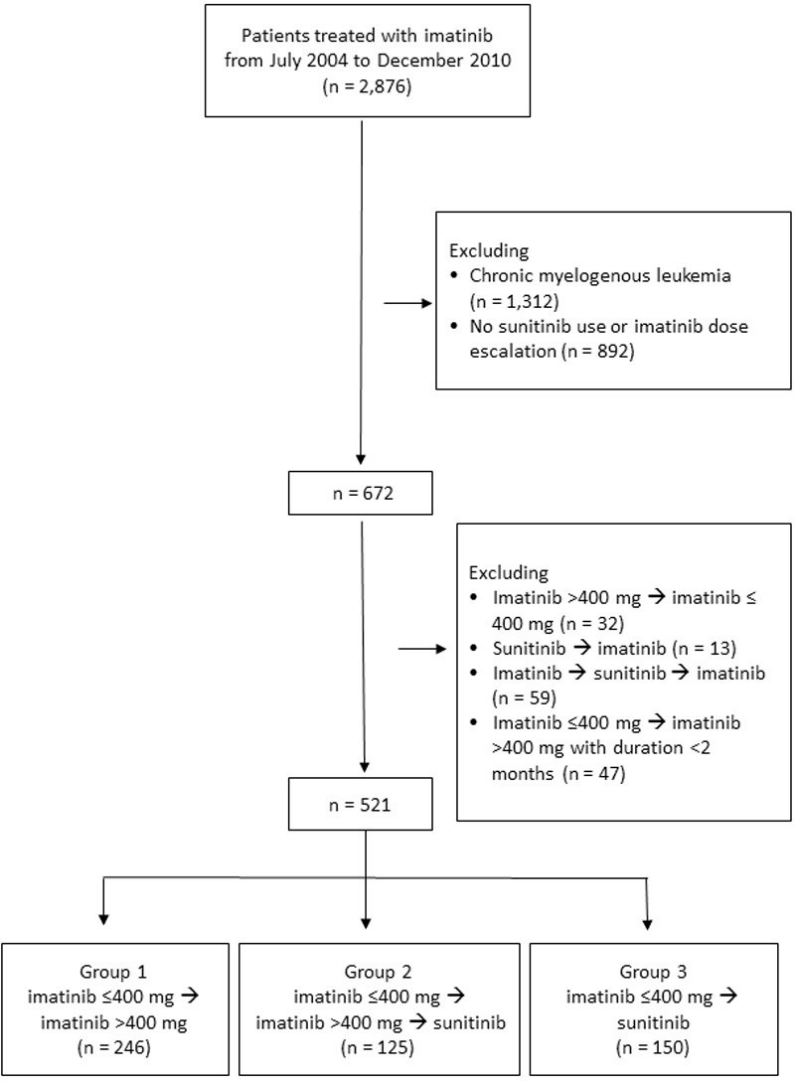
Flowchart of advanced gastrointestinal stromal tumor patients recruited in the study

We conducted a nationwide population-based cohort study using data from the Taiwan National Health Insurance Research Database (NHIRD). The NHIRD comprises healthcare data of 99.9% of the Taiwanese population, which includes sex, date of birth, place of residence, comorbidity, use of medications, managements, and diagnostic codes based on the International Classification of Diseases, 9th Revision, Clinical Modification (ICD-9-CM). The study protocol was approved by the Institutional Review Board of Chang Gung Memorial Hospital (104-6943B). The flowchart of GIST patients recruited is shown in Figure 5. Patients diagnosed with chronic myelogenous leukemia, GIST patients with stable disease (imatinib use < 400 mg/day), a treatment strategy other than imatinib (< 400 mg/day) then imatinib dose escalation (> 400 mg/day) or shift to sunitinib, and patients treated with imatinib escalation for less than 2 months were excluded in this study. Five hundred and twenty-one GIST patients facing disease progression and treated with imatinib dose escalation (> 400 mg/day) or a shift to sunitinib were enrolled in the study from July 2004 to December 2010 (Figure 5). Patients were stratified into 3 groups according to the treatment strategies: group 1, imatinib < 400 mg/day followed by imatinib dose escalation; group 2, imatinib <400 mg/day followed by imatinib dose escalation then a shift to sunitinib; group 3, imatinib <400 mg/day followed by a direct shift to sunitinib. In Taiwan, imatinib is approved and has been available for the treatment of unresectable or metastatic GIST since July 2004. Sunitinib is indicated for GIST patients with failure or intolerance to first-line imatinib treatment and has been available since February 2009. No patients received neoadjuvant or adjuvant imatinib therapy in this study (imatinib has been approved as an adjuvant since February 2011 in Taiwan). Some patients underwent debulking or salvage resections during treatment due to disease progression or tumor-related complications. The therapeutic effects of imatinib dose escalation or a direct shift to sunitinib on GIST patients and the impact of surgery on patient outcome after failure of imatinib treatment were evaluated by OS. The survival duration was calculated from the time of failure to first-line imatinib treatment (< 400 mg/day) to the last date of follow-up (December 2013) or death.

In order to track imatinib or sunitinib use, we collected data on prescriptions given and number of days supplied. The defined daily dose (DDD) was then utilized to calculate a prescribed amount of imatinib or sunitinib [25]. The DDD is the assumed average maintenance daily dose for the drug used. A cumulative DDD, which indicates both dosage and duration of drug exposure, was estimated as the total amount of prescribed imatinib or sunitinib.

### Statistical analysis

All data are presented as percentages or means with standard deviations. Numerical data were compared using the independent three-sample test. Pearson’s chi-square test and Fisher’s exact test were used for the analysis of nominal variables. Overall survival time (time-to-event) was calculated using the Kaplan-Meier method. We also utilized different models by adjusting for age, sex, Charlson comorbidity index or surgical intervention to calculate the OS.

## Abbreviations

GIST: gastro intestinal stromal tumor
OS: overall survival
PFS: progression-free survival
DDD: defined daily dose

## References

[1] Goettsch WG, Bos SD, Breekveldt-Postma N, Casparie M, Herings RM, Hogendoorn PC (2005). Incidence of gastrointestinal stromal tumours is underestimated: results of a nation-wide study. Eur J Cancer.

[2] Joensuu H, Vehtari A, Riihimäki J, Nishida T, Steigen SE, Brabec P, Plank L, Nilsson B, Cirilli C, Braconi C, Bordoni A (2012). Risk of recurrence of gastrointestinal stromal tumour after surgery: an analysis of pooled population-based cohorts. Lancet Oncol.

[3] Nilsson B, Bumming P, Meis-Kindblom JM, Odén A, Dortok A, Gustavsson B, Sablinska K, Kindblom LG (2005). Gastrointestinal stromal tumors: the incidence, prevalence, clinical course, and prognostication in the preimatinib mesylate era. Cancer.

[4] Joensuu H, Roberts PJ, Sarlomo-Rikala M, Andersson LC, Tervahartiala P, Tuveson D, Silberman SL, Capdeville R, Dimitrijevic S, Druker B, Demetri GD (2001). Effect of the tyrosine kinase inhibitor STI571 in a patient with a metastatic gastrointestinal stromal tumor. N Engl J Med.

[5] Blanke CD, Demitri GD, Von Mehren M, Heinrich MC, Eisenberg B, Fletcher JA, Corless CL, Fletcher CD, Roberts PJ, Heinz D, Wehre E (2008). Long-term results from a randomized phase II trial of standard-versus higher-dose imatinib mesylate for patients with unresectable or metastatic gastrointestinal stromal tumors expression KIT. J Clin Oncol.

[6] Demetri GD, Von Mehren M, Blanke CD, Van den Abbeele AD, Eisenberg B, Roberts PJ, Heinrich MC, Tuveson DA, Singer S, Janicek M, Fletcher JA (2002). Efficacy and safety of imatinib mesylate in advanced gastrointestinal stromal tumors. N Engl J Med.

[7] Heinrich MC, Maki RG, Corless CL, Antonescu CR, Harlow A, Griffith D, Town A, Mckinley A, Ou WB, Fletcher JA, Fletcher CD (2008). Primary and secondary kinase genotypes correlate with biological and clinical activity of sunitinib in imatinib-resistant gastrointestinal stromal tumor. J Clin Oncol.

[8] Hirota S, Isozaki K, Moriyama Y, Hashimoto K, Nishida T, Ishiguro S, Kawano K, Hanada M, Kurata A, Takeda M, Tunio GM (1998). Gain-of-function mutations of c-kit in human gastrointestinal stromal tumors. Science.

[9] Heinrich MC, Corless CL, Duensing A, McGreevey L, Chen CJ, Joseph N, Singer S, Griffith DJ, Haley A, Town A, Demetri GD (2003). PDGFRA activating mutations in gastrointestinalstromal tumors. Science.

[10] Joensuu H, Hohenberger P, Corless CL (2013). Gastrointestinal stromal tumour. Lancet.

[11] Demetri GD, van Oosteron AT, Garrett CR, Blackstein ME, Shah MH, Verweij J, McArthur G, Judson IR, Heinrich MC, Morgan JA, Desai J (2006). Efficacy and safety of sunitinib in patients with advanced gastrointestinal stromal tumour after failure of imatinib: a randomized controlled trial. Lancet.

[12] Chen YY, Yeh CN, Cheng CT, Chen TW, Rau KM, Jan YY, Chen MF (2011). Sunitinib for Taiwanese patients with gastrointestinal stromal tumor after imatinib treatment failure or intolerance. World J Gastroenterol.

[13] Vincenzi B, Nannini M, Fumagalli E, Bronte G, Frezza AM, De Lisi D, Spalato CM, Santini D, Badalamenti G, Pantaleo MA, Russo A (2016). Imatinib dose escalation versus sunitinib as a second line treatment in KIT exon 11 mutated GIST: a retrospective analysis. Oncotarget.

[14] Zalcberg JR, Verweij J, Casali PG, Le Cesne A, Reichardt P, Blay JY, Schlemmer M, Van Glabbeke M, Brown M, Judson IR (2005). Outcome of patients with advanced gastro-intestinal stromal tumours crossing over to a daily imatinib dose of 800mg after progression on 400mg. Eur J Cancer.

[15] Rankin C, Von Mehren M, Blanke C, Benjamin R, Fletcher CD, Bramwell V, Crowley J, Borden E, Demetri GD (2004). Dose effect of imatinib (IM) in patients with metastatic GIST-Phase III Sarcoma Group Study S0033. J Clin Oncol.

[16] Hsu CC, Wu CE, Chen JS, Tseng JH, Chiang KC, Liu YY, Tsai CY, Cheng CT, Chen TW, Jan YY, Yeh TS (2014). Imatinib escalation or sunitinib treatment after first-line imatinib in metastatic gastrointestinal stromal tumor patients. Anticancer Res.

[17] (2010). Comparison of two doses of imatinib for the treatment of unresectable or metastatic gastrointestinal stromal tumors: a meta-analysis of 1,640 patients. J Clin Oncol.

[18] Hislop J, Mowatt G, Sharma P, Raser C, Elders A, Jenkinson D, Vale L, Petty R (2012). Systematic review of escalated imatinib doses compared with sunitinib or best supportive care, for the treatment of people with unresectable/metastatic gastrointestinal stromal tumours whose disease has progressed on the standard imatinib dose. J Gastrointest Cancer.

[19] Casali P, Blay JY (2010). Gastrointestinal stromal tumours: ESMO clinical practice guidelines for diagnosis, treatment and follow-up. Ann Oncol.

[20] Demetri GD, Von Mehren M, Antonescu CR, DeMatteo RP, Ganjoo KN, Maki RG, Pisters PW, Raut CP, Riedel RF, Schuetze S, Sundar HM (2010). NCCN Task Force report: update on the management of patients with gastrointestinal stromal tumors. J Natl Compr Canc Netw.

[21] Guidelines for the management of gastrointestinal stromal tumours (GISTs) (2005). http://www.augis.org/news_guidelines/management_%20guidelines.htm.

[22] Rubió-Casadevall J, Martinez-Trufero J, Garcia-Albeniz X, Calabuig S, Lopez-Pousa A, Del Muro JG, Fra J, Redondo A, Lainez N, Poveda A, Valverde C (2015). the Spanish Group for Research on Sarcoma (GEIS). Role of surgery in patients with recurrent, metastatic, or unresectable locally advanced gastrointestinal stromal tumors sensitive to imatinib: a retrospective analysis of the Spanish Group for Research on Sarcoma (GEIS). Ann Surg Oncol.

[23] Park SJ, Ryu MH, Ryoo BY, Park YS, Sohn BS, Kim HJ, Kim CW, Kim KH, Yu CS, Yook JH, Kim BS (2014). The role of surgical resection following imatinib treatment in patients with recurrent or metastatic gastrointestinal stromal tumors: results of propensity score analyses. Ann Surg Oncol.

[24] Yeh CN, Chen TW, Tseng JH, Liu YY, Wang SY, Tsai CY, Chiang KC, Hwang TL, Jan YY, Chen MF (2010). Surgical management in metastatic gastrointestinal stromal tumor (GIST) patients after imatinib mesylate treatment. J Surg Oncol.

[25] WHO Collaborating Centre for Drug Statistics Methodology: ATC/DDD Index 2016. In http://www.whocc.no/atc_ddd_index/. Accessed October 13, 2016

